# Technologies and Strategies for Metabolic and Molecular Imaging With Hyperpolarized MRI

**DOI:** 10.1002/jmri.70084

**Published:** 2025-09-01

**Authors:** Alixander S. Khan, Christoffer Laustsen

**Affiliations:** ^1^ MR Research Centre, Department of Clinical Medicine Aarhus University Aarhus Denmark

**Keywords:** d‐DNP, hyperpolarization, PHIP, SEOP

## Abstract

Conventional Magnetic Resonance Imaging (MRI) offers limited sensitivity for direct metabolic and molecular imaging using non‐proton nuclei due to low thermal nuclear spin polarization. Hyperpolarization (HP) technologies increase nuclear spin polarization by several orders of magnitude, overcoming this limitation to enable in vivo studies of biochemistry and physiology. A growing body of literature has shown the value in HP technologies offering metabolic and functional information useful for a variety of clinical applications. This review details the complete workflow of hyperpolarized MRI, from the methodologies used to produce hyperpolarized samples and the range of available probes to the specific imaging acquisition and data analysis strategies required to maximize the signal, and finally, its major clinical applications.

**Evidence Level:**

5.

**Technical Efficacy:**

Stage 3.

## 1 Introduction

Magnetic Resonance Imaging (MRI) plays a vital role in diagnosis where structural changes are assessed and associated with disease and clinical conditions. The high natural abundance of ^1^H alongside its relatively high magnetic moment means it is conventionally used for structural and advanced MR methods [1]. However, MRI can go beyond structural measurement with measurement of physiological information using other nuclei to obtain biological function. While ^1^H can be used, low concentrations in relevant biomolecules make measurement difficult [2]. Alternative nuclei are attractive for physiological measurements, but low natural abundance and MR signal impact their MRI use. Hyperpolarization is a technology that allows for a significant increase in the sensitivity of MRI by increasing the nuclear spin polarization, thereby boosting the MR signal strength. Through this, alternative nuclei can be imaged after going through the hyperpolarization process, allowing for a wide range of molecules to be applied to imaging studies. This review details hyperpolarization processes, probes used in HP studies, methods for imaging acquisition, subsequent data analysis strategies, and the clinical applications of hyperpolarized MRI.

## 2 Fundamentals of Hyperpolarization

**FIGURE 1:**
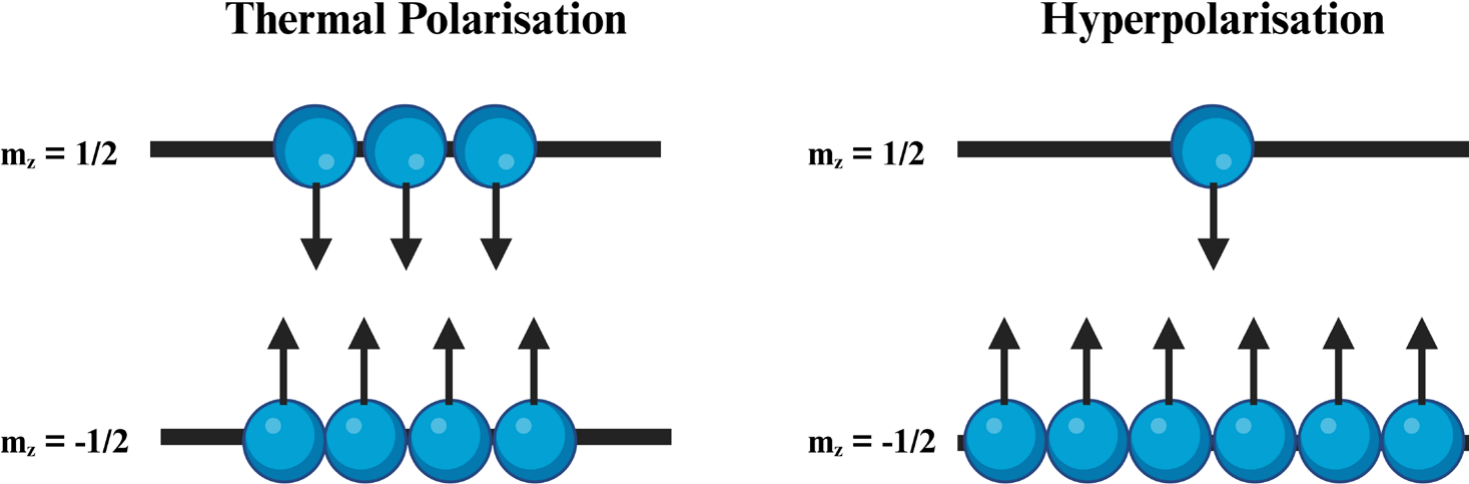
Polarization of spin states at both thermal polarization and following hyperpolarization. Thermal polarization produces a small excess in one spin state, producing a lower MR signal compared to hyperpolarization, where a much larger excess exists.

The MR signal arises from a population difference between nuclear spin energy states. This nuclear spin polarization, an excess of nuclei in one spin state, contributes to the MR signal. However, at thermal polarization, only a small fraction of spins is in excess and contributes to the MR signal (Figure 1). The net population difference between the two spin states can be given by the Equation (1).

(1) $$P=\;\tanh\left(\frac{\left(\hbar \gamma B_{0}\right)}{\left(2k_{B}T\right)}\right)$$

where ℏ is Planck's constant, 𝛾 is the gyromagnetic ratio, 𝐵0 is the magnetic field, 𝑘𝐵 is Boltzmann's constant, and 𝑇 is temperature. Whilst nuclei with higher gyromagnetic ratios offer increased MR signal, altering either temperature or magnetic field can increase net polarization. Therefore, for the MR signal to be increased, either the temperature must be reduced or the magnetic field must be increased. Hyperpolarization methods utilize the equation to increase the alignment of nuclei's spin. Prior to MR imaging, various techniques produce hyperpolarized samples, increasing polarization over 10,000‐fold from ~0.00025% (thermal) to 30%–40% [3].

The polarization of spin states is impacted by spin–lattice relaxation (T_1_) where over time the polarization decays exponentially to return to thermal polarization levels. Whilst T_1_ is often long‐lived at low temperatures and high magnetic fields, when at clinical settings it can significantly shorten, limiting the samples that can be used for imaging, and the time frame in which imaging can be completed. Additionally, the polarized state is non‐renewable; RF pulses convert longitudinal polarization to detectable transverse magnetization, consuming that portion of the hyperpolarized state and causing signal decay as the spin state is no longer polarized after excitation. The effect of repeated RF pulses on a polarized sample can be described by Equation (2).
(2)

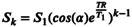

where *S*_*k*_ is the transverse signal of the polarized sample after the *k*th excitation, *S*_1_ is the first transverse signal, and *α* is the flip angle. Thus, imaging techniques require careful consideration, as excessive flip angles or frequent RF pulses cause rapid signal decay.

### 2.1 Hyperpolarized Techniques

There are several methods for hyperpolarization with varying technologies requiring differing equipment and timescales for effective sample production. This section will briefly review the five main methods to produce hyperpolarized samples.

#### 2.1.1 Brute Force Polarization

Brute Force polarization offers a simple method of polarization where a sample is exposed to low temperature and high magnetic field before being extracted after some time [4]. In doing so, a hyperpolarized sample can be produced without the need for radicals or microwave excitation, avoiding the need for filters and more complex technologies [5]. Whilst this has been applied to samples such as ^129^Xe and [1‐^13^C] pyruvic acid, the need for considerable time (up to several days) to achieve a useful polarization value limits the application of this technique.

#### 2.1.2 Dissolution—Dynamic Nuclear Polarization (d‐DNP)

**FIGURE 2:**
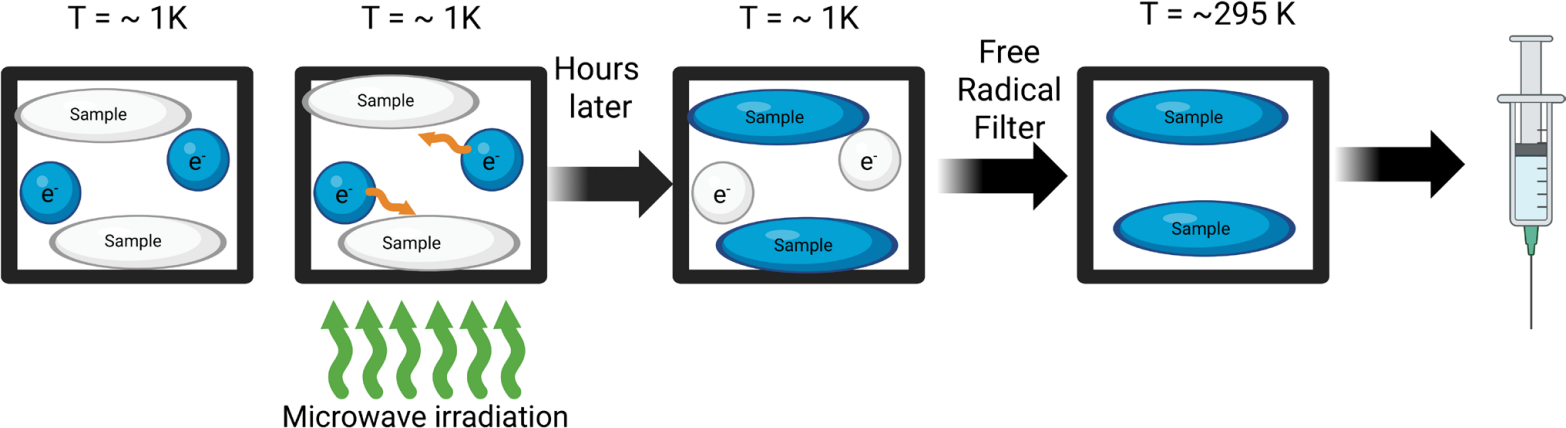
Process of DNP: ^13^C‐pyruvate is with electrons (free radicals) at low temperatures (~1 K). Microwave irradiation is applied; and after some time, the hyperpolarization is transferred to the ^13^C pyruvate (indicated by the changing color of the ^13^C pyruvate and the electrons). Afterwards the sample is heated to room temperature (*T* = 295 K) and a radical filter is applied to remove the electrons from the sample.

Dynamic nuclear polarization (DNP) is the most common method for producing liquid hyperpolarized samples. This process relies on utilizing free radical electrons high polarization when at low temperature and high magnetic field and transferring the polarization to a target nuclei [3]. The polarization transfer uses the Overhauser effect [6] by applying microwaves near the electron Larmor frequency. As a result, nuclei polarization increases as polarization transfers, leading to solid‐state polarized sample after a period of hours. The most important part of the d‐DNP process for applications to imaging is the dissolution process in order to produce a clinically usable sample while sustaining the polarization. The dissolution process requires a rapid (< 5 s) and sterile injection of hot solvent to melt the frozen sample and flush it from the polarization chamber to be able to be injectable [7]. Radical filtration must be offered for in vivo use, requiring validated filters and QC methods to ensure residual radical concentrations are below toxicological limits (Figure 2). This entire process, in particular for clinical use, requires significant infrastructure beyond a standard MRI setup, including a dedicated polarizer unit containing a low‐temperature (~1 K) high‐field magnet (typically 3–7 T), a high‐power microwave source (~100 GHz), and cryogen handling (liquid helium). For clinical translation, a GMP‐compliant automated fluid path for dissolution, quality control (QC), and injection is essential, as exemplified by the commercial SPINlab system. Several substrates can be polarized in this fashion but rely on the lifetime being long enough for the polarization to be successful. The HP lifetime is determined by the nuclear T1, with the hyperpolarized signal enhancement decaying to approximately 5% of its initial value after a time equivalent to three times T_1_. Recently, attempts have been made to extend the T_1_ by adding additional solvents to the sample such as D_2_O [8] and gadolinium [9].

#### 2.1.3 Parahydrogen Induced Polarization (PHIP)

**FIGURE 3:**
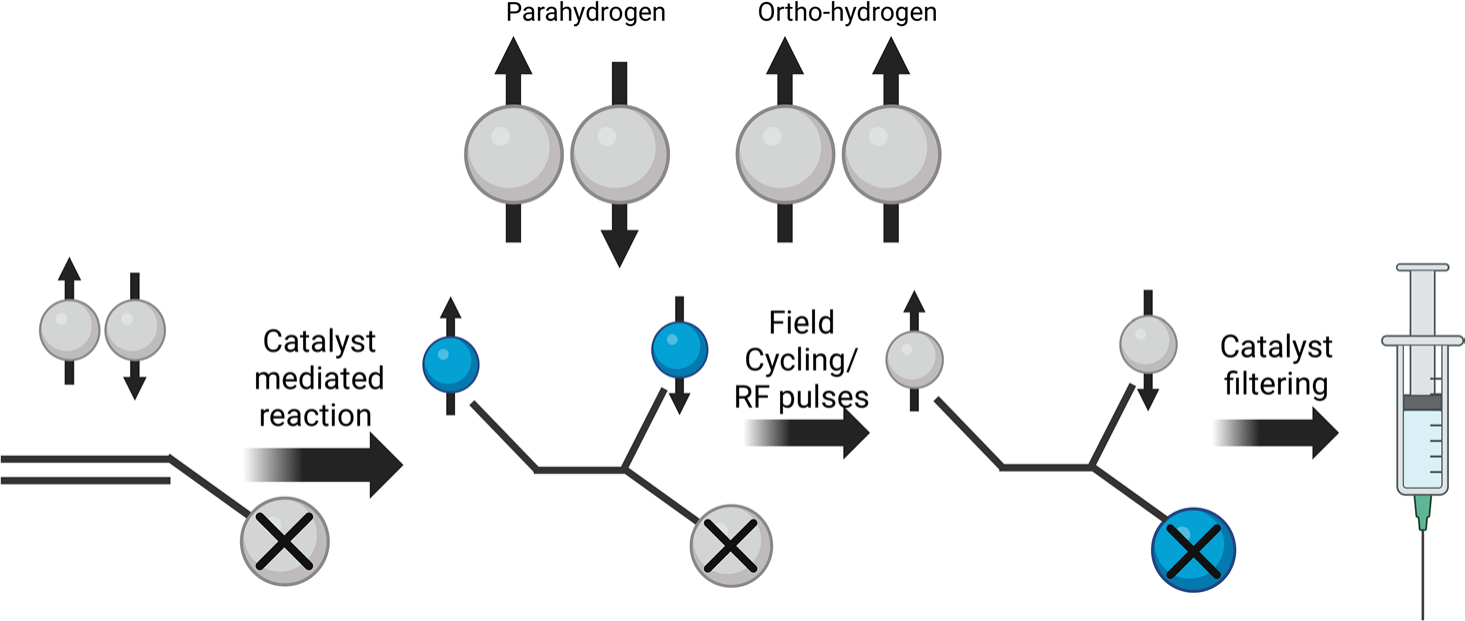
Process of PHIP wherein parahydrogen binds with an unsaturated molecule via a catalyst‐mediated reaction breaking the parahydrogen's singlet state and producing a polarized ^1^H molecule. Field cycling and RF pulses can allow for the polarization to be transferred to an alternative nucleus (denoted X). Catalyst filtering is required to produce a safe polarized hyperpolarized probe.

PHIP uses a method of adding para‐hydrogen to a substrate to create a hyperpolarized molecule, taking advantage of the multiple spin states of hydrogen. Hydrogen has four different spin states that, at room temperature, have equal probability of occupying. Three of these are triplet states with a net spin of 1 (called ortho‐hydrogen) and one spin state with a singlet state where the net spin is 0 (called para‐hydrogen). The net spin of para‐hydrogen being 0 means that it is “radio silent”, that is, it is invisible in NMR. However, when para‐hydrogen is added to an unsaturated substrate, the symmetry of the singlet state can be broken, producing a hyperpolarized ^1^H sample (Figure 3). To add para‐hydrogen to the substrate, catalytic hydrogenation must be performed using rhodium or iridium as a catalyst. For the polarization process to be performed, para‐hydrogen needs to be created without the presence of ortho‐hydrogen, which exists at a 3:1 ratio at room temperature. Since para‐hydrogen is a lower energy state, by decreasing the temperature, the population of para‐hydrogen can be increased and becomes the dominant form at ≈30 K. However, this process can take considerable time and becomes a costly process at the low temperatures required. Instead, the production can incorporate a catalyst such as metal oxides or activated carbon to help alleviate these problems by speeding up the process. By using a catalyst, the production time is dramatically reduced, enabling para‐hydrogen to be produced in large quantities within hours.

This process enables hyperpolarized ^1^H MRI [10], but it lacks physiological information from other nuclei and suffers from short T_1_. As a result, the polarization is able to be transferred from ^1^H to other nuclei through methods such as field cycling [11] or specialized pulse sequences [12]. However, despite this the application is limited since it requires structural modifications [13] which limits biomedical applications. Recent advances have allowed PHIP to applied to a wider range of molecules using precursors with a side arm capable of hydrogenation to produce hyperpolarized products. This development, side arm hydrogenation (SAH) has allowed for the development of a wider range of biologically useful polarized products such as [1‐^13^C] pyruvate [14], [1‐^13^C] acetate [15] and [1,4‐^13^C_2_] fumerate [16, 17].

Whilst PHIP‐SAH allows for a wider range of polarized products, the method suffers from lower polarization values compared to d‐DNP techniques, with the highest polarization value being 18% [14]. In addition, the use of a catalyst in the first stage of the PHIP process introduces additional safety considerations. Residual catalyst (often heavy metals like Rh or Ir) must be rigorously removed to nanomolar levels prior to injection, demanding stringent purification protocols and sensitive QC. However, recent developments have meant that both polarization levels have increased, with results comparable to d‐DNP, and contaminants decreased [14] allowing for easier translation for clinical investigations [14]. As a result of its easier polarization process, potential translation for biomedical spin‐outs exists, with nVision and magnikeen offering translational opportunities.

#### 2.1.4 Signal Amplification by Reversible Exchange (SABRE)

**FIGURE 4:**
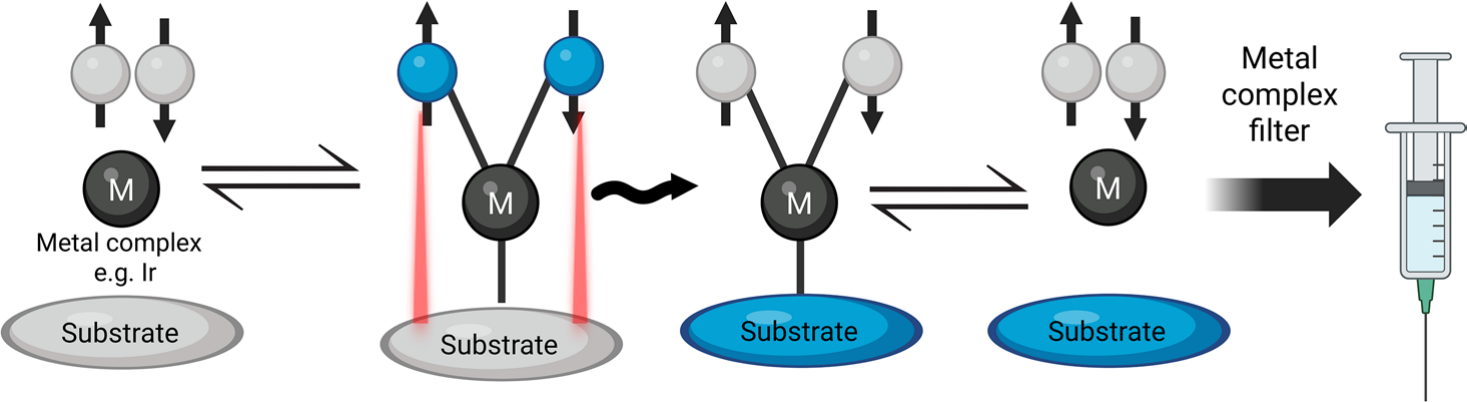
Process of SABRE. A metal complex is used to facilitate the polarization transfer from the parahydrogen to the substrate of interest. The parahydrogen and substrate attach to the metal complex and polarization is transferred via J‐coupling (denoted by red) before the substrate and parahydrogen detach from the metal complex. A filter is then applied to remove the metal complex from the sample, however a fraction still remains making application to the clinic difficult.

SABRE expands on PHIP concepts, using a metal complex [18] to bind parahydrogen and substrate. Spin–spin coupling via J‐couplings then polarizes the substrate with similar mechanisms to PHIP. To transfer the polarization, there are distinct requirements for the design of the metal catalyst. Firstly, the bonds need to be effective at transferring the polarization between para‐hydrogen and the substrate. This is achieved via a J‐coupling network that transfers the polarization via the metal center [19] (Figure 4). Secondly, the metal complex's kinetics must match the kinetic requirements of SABRE. The kinetics of the metal complex is determined by its exchange rate of the two attachments. The exchange rate must be sufficiently large that the metal complex is able to act on lots of substrates for the net polarization of the system to increase. If this can be achieved, the “bulk” polarization decay can be ignored since the overall level of polarization is able to be increased. However, if the exchange rate is too large then the substrate will not be attached for long enough for the polarization to be transferred properly. Alternatively, if the exchange rate is too small, then the substrate will attach to the complex for too long and relaxation processes will begin to be introduced. Whilst several metal complexes can fulfill the first requirement of establishing a J‐coupling network, very few match the requirements of the second. The most successful catalyst was found to be an Iridium (Ir)‐based catalyst [20] with an exchange rate in the order of tens of Hz. The Ir metal complexes have a metal center, but the surrounding chemical identity can be varied to match the requirements of the substrate to be polarized. A range of molecules have been shown to be polarized with SABRE, such as [1‐^13^C] pyruvate for metabolic studies or tuberculosis medication [21] offering the potential for drug tracer studies. In addition, recent advances in SABRE production have meant that the level of toxic solvent and iridium was low enough for in vivo pre‐clinical studies whilst also having sufficient polarization for imaging [22]. Whilst this remained significantly too high for clinical applications, there remain possibilities for SABRE applications in pre‐clinical studies for low cost polarization investigations (Figure 4).

#### 2.1.5 Spin Exchange Optical Pumping (SEOP)

**FIGURE 5:**
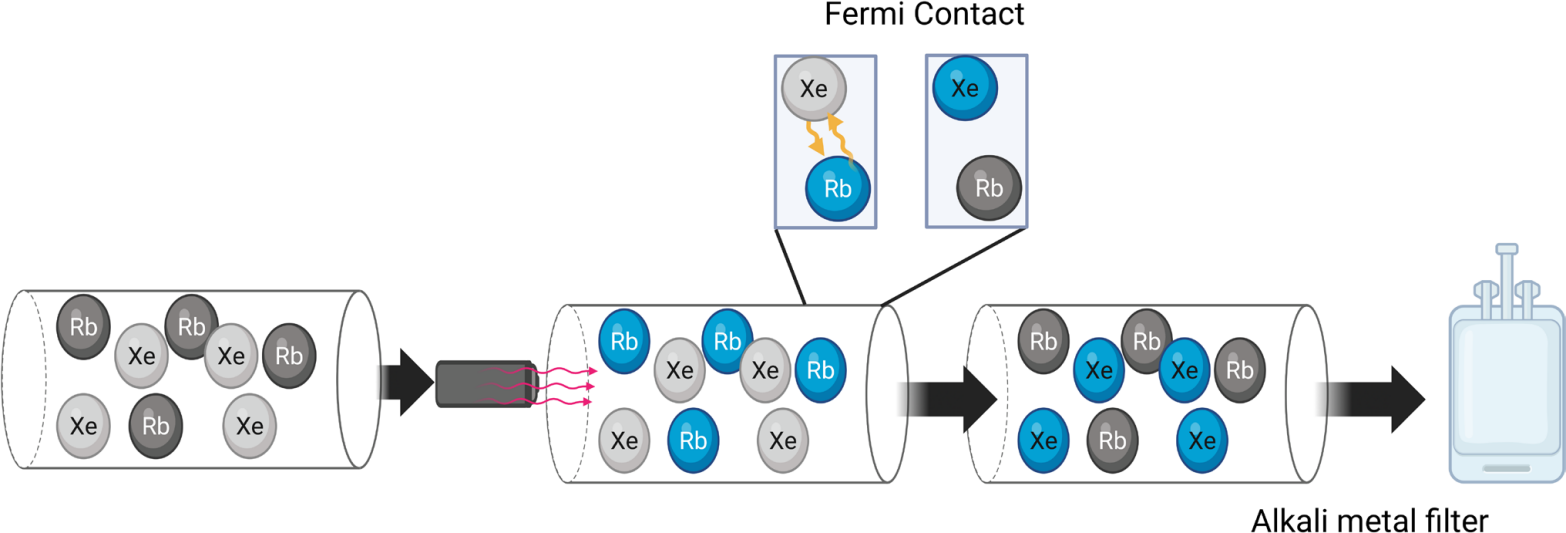
Process of SEOP: A gas, in this case ^129^Xe is placed in a glass cylinder alongside a vaporized alkali metal. Circularly polarized light is applied to the glass cell at a resonant frequency to create a spin‐polarized alkali metal which in turn transfers the polarization to the ^129^Xe via Fermi contact. An alkali metal filter is then applied to create a pure hyperpolarized gas sample.

SEOP is a method for the hyperpolarization of noble gases (e.g., ^3^He, ^129^Xe). Through this process, alkali metals become electronically spin‐polarized in the presence of a magnetic field and circularly polarized light, and polarization is subsequently transferred to noble gas atoms via gas‐phase collisions (Figure 5) [23]. The principle of SEOP can be broken into two stages. In the first stage, an alkali metal is vaporized by setting the system to a high temperature (≈120°C–180°C) within a glass cell. Circularly polarized light is then applied to the vaporized metal whilst in the presence of a weak magnetic field (≈50 G). The laser is tuned to the alkali's resonance frequency, which drives a transition of the metal's electrons' spins. Within the glass cell, the noble gas is also present. The spin‐polarized alkali metal transfers the polarization to the noble gas via Fermi‐contact hyper‐fine interactions. These interactions lead to the noble gas nuclei entering a polarized state whilst the alkali metal loses its polarization and can then be polarized again by re‐absorption of the laser photons [24]. A variety of different alkali metals can be used for SEOP, with most using rubidium (Rb) [25]. An important feature of Rb is its absorption wavelength, which corresponds with many commercially available lasers, at 794.77 nm. However, the practical implementation involves considerable technical challenges, including the safe handling of reactive alkali metals (like Rb), maintaining high gas purity, stabilizing high‐power lasers tuned precisely to the alkali D1 transition, and mitigating polarization losses due to interactions with the container walls. To further improve the polarization process, a buffer gas is used to increase the efficiency of the polarization and reduce energy loss. The addition of a buffer gas acts to pressure broaden the alkali's absorption wavelength and therefore increase the number of photons absorbed by the alkali metal. The buffer gas also serves to prevent alkali metal fluorescence, which occurs when the alkali metal relaxes back to the ground state. This prevents the loss of energy and ensures that a high amount of polarization is transferred to the noble gases. Most commonly, ^14^N is used as a buffer gas, offering both the ability to pressure broaden the system and quench the fluorescence of alkali metals. SEOP can follow two methods of production: batch‐mode or continuous flow [26]. Through these two techniques, whilst the overall methodology remains the same, differences in production result in different equipment and production capabilities. In batch‐mode, a fixed quantity of gas is loaded into the chamber before the SEOP process begins for a set period to produce hyperpolarized samples that can be used. In comparison, continuous flow involves a xenon gas flow through the SEOP cell before being cryo‐collected in a solid state, allowing for greater volumes to be produced. Hyperpolarized ^129^Xe has achieved FDA clearance as a contrast agent, with several commercial SEOP existing using both stopped and continuous flow methods.

### 2.2 Hyperpolarized Probes

Several probes can be produced using the hyperpolarized technologies discussed. However, the probes must be biologically relevant for in vivo imaging while capable of being polarized to sufficient levels for effective imaging. The delivery of the probes can be divided into two options: injection of a hyperpolarized liquid or inhalation of a hyperpolarized gas. Hyperpolarized probes have several requirements, both technically to produce useful information and biologically to ensure safe in vivo.

### 2.3 Ventilation Probes

Hyperpolarized gas relies on the inhalation of the probe for imaging studies. Traditional lung imaging methods have been compounded by the low signal due to limited density within the organ, and the provision of an external probe offers an alternative method for imaging. In addition, gas probes can be inhaled, and the hyperpolarized probe can be transferred to other organs for functional imaging. For the hyperpolarized probe to be successful, it must exist in gaseous form with sufficient T_1_ and the ability to be polarized by SEOP.

#### 2.3.1 Helium‐ ^3^He

The high gyromagnetic ratio of ^3^He (32.4 MHz T^−1^) and inert nature makes it an attractive probe for gaseous imaging. Initial hyperpolarized gas studies showed the feasibility of the imaging approach with high spatial resolution and clinically useful images [27]. Imaging studies focused on ventilation maps offering a spatial measure of defects in the lung [28]. Whilst a high signal is able to be produced, ^3^He is effectively insoluble in blood and tissues [29] limiting measurements to only lung gas images. In addition, ^3^He has become more costly due to worldwide shortages, making widespread use for clinical studies difficult.

#### 2.3.2 Xenon— ^129^Xe

**FIGURE 6:**
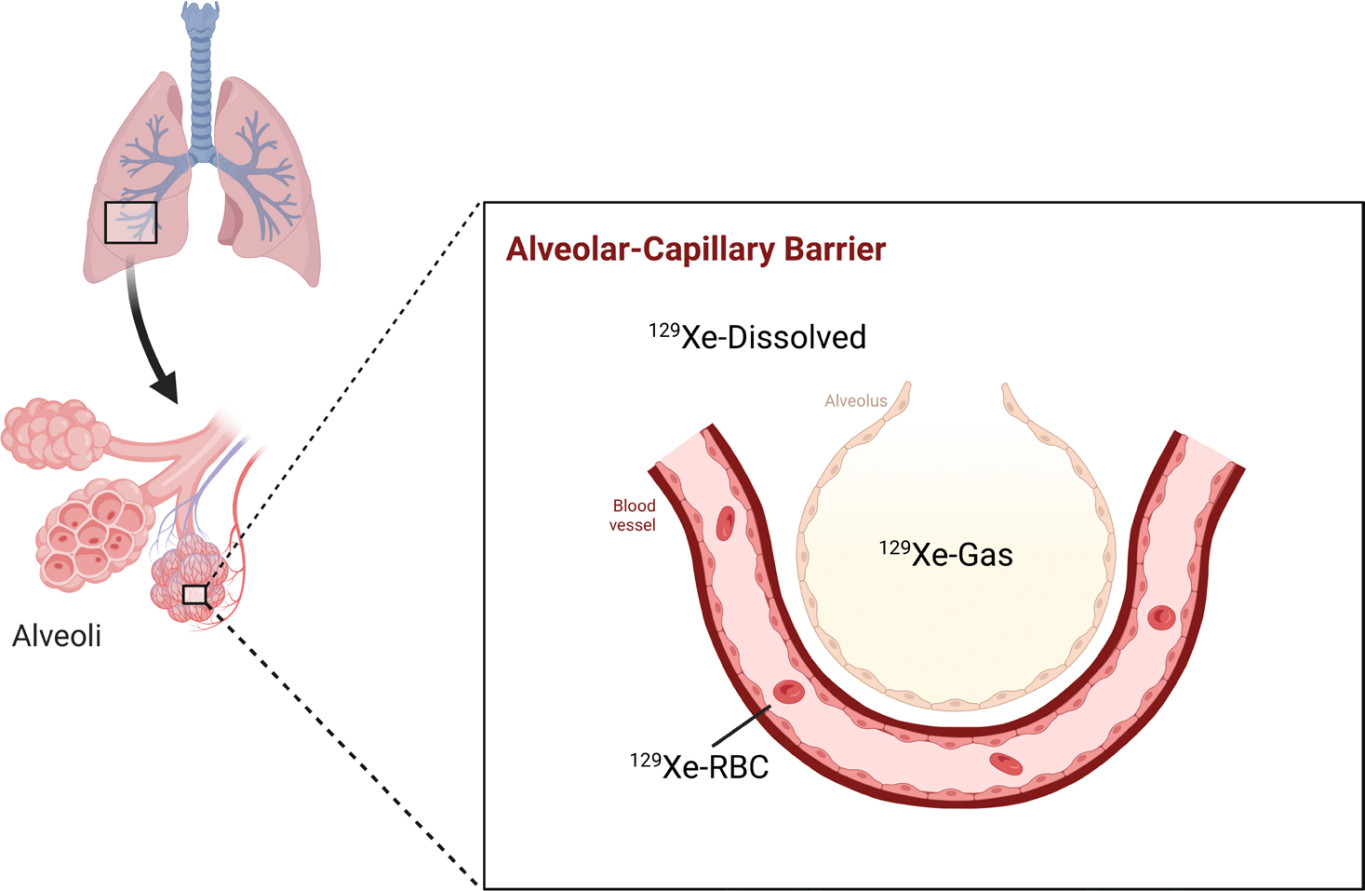
Hyperpolarized ^129^Xe for lung imaging allows for ventilation imaging through measurement of distribution across the lungs as well as measurement of the gas uptake into blood and tissue. ^129^Xe is absorbed through the alveoli to the bloodstream and tissue, offering a measure of uptake that can be disturbed in disease.

Whilst ^129^Xe has a lower gyromagnetic ratio (11.8 MHz T^−1^) making the signal lower than ^3^He, the hyperpolarization process results in a sufficient signal for imaging studies. Application to the lung allows for ventilation‐based imaging in a similar form to ^3^He but is further enhanced by the lipophilic properties where the gas is soluble in tissue and blood [30]. In doing so, the application can be extended beyond ventilation imaging where ^129^Xe can be exchanged from the lungs to different compartments with different chemical shifts [31] (gaseous ^129^Xe = 0 ppm, Red Blood Cell (RBC) ^129^Xe = 218 ppm, Blood Plasma/Tissue ^129^Xe = 197 ppm). As such, further measurements such as gas perfusion rate can be measured from the uptake of ^129^Xe into the lung vascular (Figure 6). However, this is impeded by the significant shortening of T1 relaxation times once the gas dissolves into blood and tissue [32], rapidly diminishing the available signal for downstream perfusion or uptake measurements. Whilst the standard method of production for HP ^129^Xe uses SEOP, d‐DNP has also been applied to ^129^Xe [33, 34] achieving levels as high as 30%.

### 2.4 Liquid Probes

Hyperpolarized liquid probes, produced via d‐DNP, PHIP, or SABRE, are designed to be injected into the body prior to MRI acquisition to probe biochemical processes in the body. Several diseases within the body trigger biochemical changes that, if probed, can allow for the disease to be better understood using MRI.

#### 2.4.1 Metabolic

A particular focus for hyperpolarized imaging has been metabolic imaging. Metabolism plays a vital part in normal physiology, and changes are often some of the first markers of disease. Whilst different organs have different rates of metabolic activity and downstream production, all mainly rely upon glucose as the primary energy source. Whilst the obvious choice for a hyperpolarized probe would be glucose, the short T_1_ and complex metabolic fate limit its use. Instead, downstream metabolic products are chosen, with the choice depending on providing the metabolic pathway it can probe. Several hyperpolarized capable nuclei have been considered; however, ^13^C is often chosen owing to its low natural abundance within the body, ability to achieve sufficiently high polarization, and biological relevant molecules.

#### 2.4.2 Pyruvate ([1‐ ^13^C] Pyruvate and [2‐ ^13^C] Pyruvate)

**FIGURE 7:**
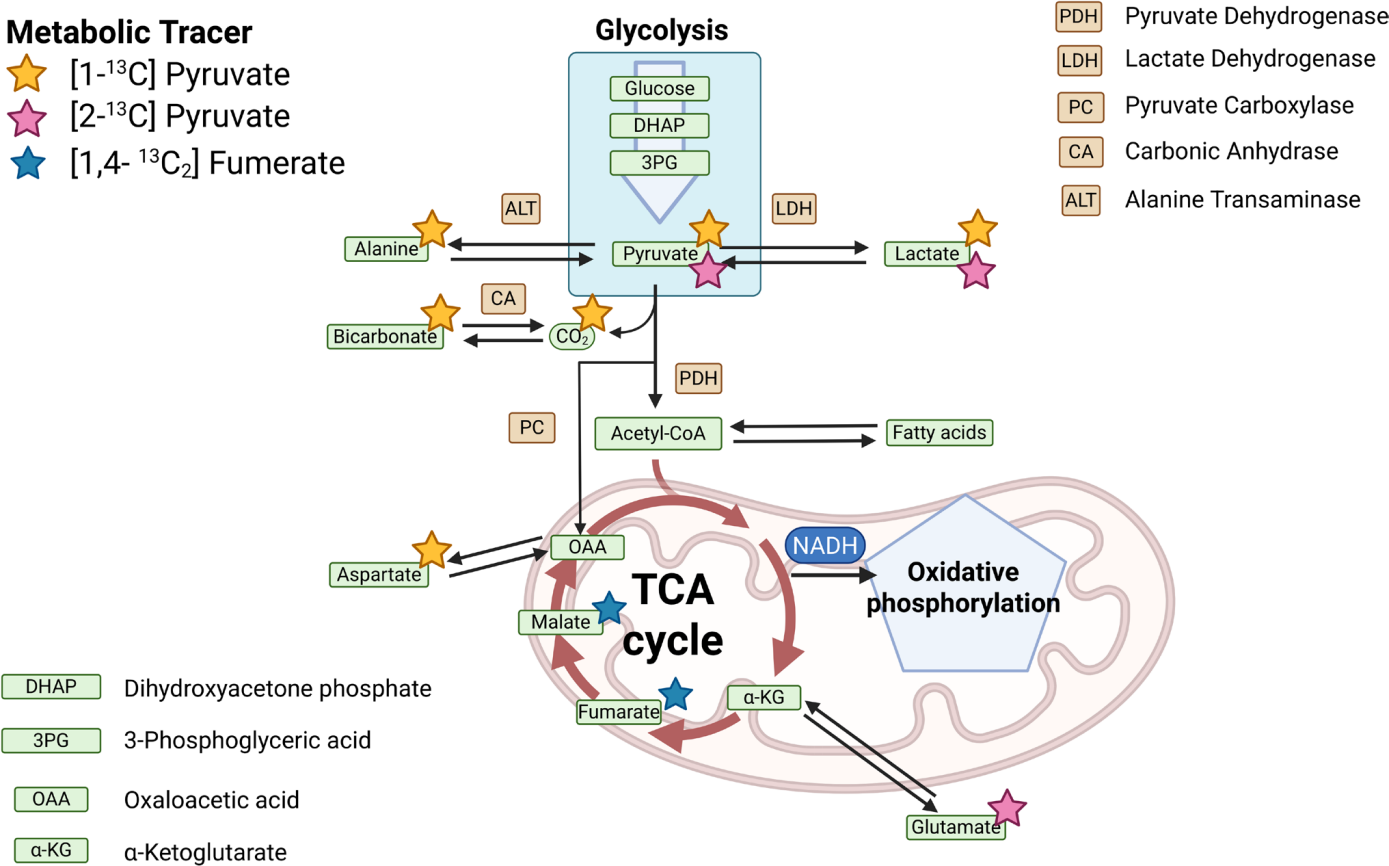
Overview of metabolism that can be probed with hyperpolarized tracers. Pyruvate lies at an important fate of metabolism with several different products offering insights into several enzymes. The labeling position of choice determines the metabolites able to be measured. Fumarate and malate are two important intermediaries in the TCA cycle where measurement offers an insight into cellular death.

Pyruvate lies at a vital cross section of metabolism, playing a key role as an intermediary for metabolic products. As a result, its use as a metabolic probe is of great utility, with the production of downstream products informing on several metabolic pathways. Following injection of pyruvate, vascular transportation results in delivery to organs where extracellular pyruvate enters the cells via monocarboxylate transporters (MCT) for metabolic conversion (Figure 7). The subsequent metabolic products rely on the choice of carboxyl labeling, with different positions resulting in different pathways able to be traced.

##### 2.4.2.1 [1‐ ^13^C] Pyruvate

[1‐^13^C] pyruvate is the most common metabolic tracer in research studies with application to the brain, heart, kidney, liver, and prostate. Following the transportation of pyruvate into the cytosol, enzymatic conversion can produce four detectable metabolic byproducts with distinguishable ppm values to allow for independent measurements of metabolites ([1‐^13^C] pyruvate = 171 ppm, ^13^C‐Lactate = 181 ppm, ^13^C‐Alanine = 178 ppm, [1‐^13^C] aspartate = 175 ppm, ^13^C‐bicarbonate = 162 ppm). Within the cytosol, reaction with LDH produces ^13^C‐lactate, and ALH produces ^13^C‐alanine. Alternatively, pyruvate can be transported into the mitochondria via the mitochondria pyruvate carrier (MPC) where it can undergo metabolic conversion and enter the TCA cycle. Through this process, the PDH enzymatic reaction occurs with acetyl CoA, with ^13^CO_2_ produced as a byproduct, which enters rapid equilibrium with bicarbonate because of carbonic anhydrase to produce an MR detectable ^13^C‐bicarbonate. In doing so, mitochondrial metabolism can be assessed with ^13^C‐bicarbonate (H^13^CO_3_^−^) offering a readout of PDH activity. Finally, ^13^C‐aspartate can be detected following the incorporation of ^13^CO_2_ via pyruvate carboxylase (PC) or from the back conversion of [1‐^13^C] oxaloacetic acid to malate to fumarate and subsequently aspartate. However, while detected in both the liver and the brain, the measurement of this path remains limited due to low SNR because of the lower rate of PC activity.

##### 2.4.2.2 [2‐ ^13^C] Pyruvate

Whilst PDH information can be obtained from [1‐^13^C] pyruvate, further information can be obtained from [2‐^13^C] pyruvate where the 2‐position carbon remains on acetyl CoA and enters the TCA cycle [35]. Through this, [2‐^13^C] lactate can be detected allowing for LDH measurement as well as [5‐^13^C] glutamate where it has been applied to the brain, liver, and heart with sufficient ppm separation for accurate quantification ([2‐^13^C] pyruvate = 207.8 ppm, [5‐^13^C] glutamate = 183.9 ppm, [2‐^13^C] lactate = 71.3 ppm). Whilst offering greater metabolic information, the presence of heteronuclear and homonuclear J‐couplings involving the ^13^C‐2 position complicates spectral quantification [36]. As such, more advanced acquisition and processing techniques are required compared to the relatively simpler spectrum of [1‐^13^C] pyruvate and its products.

#### 2.4.3 [1,4‐ ^13^C_2_ ] Fumarate

Fumarate lies within the TCA, playing an important role as an intermediary within the cell (Figure 7). In healthy conditions, fumarate is converted to malate by the enzyme fumarase intracellularly. However, in the case of cell death, fumarase is released into the extracellular space, creating extracellular malate. Injection of hyperpolarized ^13^C‐fumarate offers the potential to measure cell death through the measurement of [1,4‐^13^C_2_] fumarate (176.0 ppm) to [1,4‐^13^C_2_] malate (182.5, 181.4 ppm). Application of hyperpolarized fumarate has been limited to pre‐clinical studies, with both d‐DNP and PHIP showing the potential for hyperpolarized imaging in kidney disease [37], cancer models [38] and cardiovascular disease [39] with recent work to develop it for clinical application [40].

### 2.5 Perfusion

Perfusion based changes reflect vascular changes that are important for disease monitoring. Whilst perfusion‐based measurements using techniques such as dynamic contrast enhanced imaging, the use of gadolinium remains a concern for routine use. Metabolically inactive probes can be used for tracing uptake and allow for dynamic measurement for perfusion‐based imaging.

#### 2.5.1 ^13^C‐Urea

^13^C‐urea is a safe and biologically inert tracer suitable for perfusion measurements [41, 42, 43, 44, 45]. The use of ^13^C‐urea can be enhanced through ^15^N labeling to increase the T_2_ relaxation, allowing for SNR gains when applied using bSSFP imaging [46]. Co‐polarization of [1‐^13^C] pyruvate and [^13^C, ^15^N_2_] urea allows for metabolic and perfusion assessment in the same scan [42] and application has been shown in pre‐clinical renal disease [43], cancer models [44] and healthy human brains [45].

#### 2.5.2 ^13^C‐HP001

HP001, *bis*‐1,1‐(hydroxymethyl)‐[1‐^13^C] cyclopropane‐d_8_, offers another ^13^C‐molecule for perfusion studies that is biologically inactive and offers sufficient T_1_ values for polarization studies. Application to myocardial perfusion quantification showed the potential for improved perfusion measurements compared to DCE measurements [47] but suffers from not being an endogenous compound in humans, making clinical translation difficult [48].

#### 2.5.3 ^1^H_2_O

Hyperpolarization of H_2_O offers an attractive HP tracer owing to high potential signal due to the gyromagnetic ratio of ^1^H and the high concentration of water in tissue, alongside an excellent safety profile. Polarization studies [49] have shown the ability to produce polarized samples with a continuous flow method employed to optimize imaging. Injection of the hyperpolarized water into pre‐clinical models has shown the ability to detect the tracer [50] and validated the respective perfusion measurements with gadolinium‐based measurements [51].

#### 2.5.4 129‐Xenon

**FIGURE 8:**
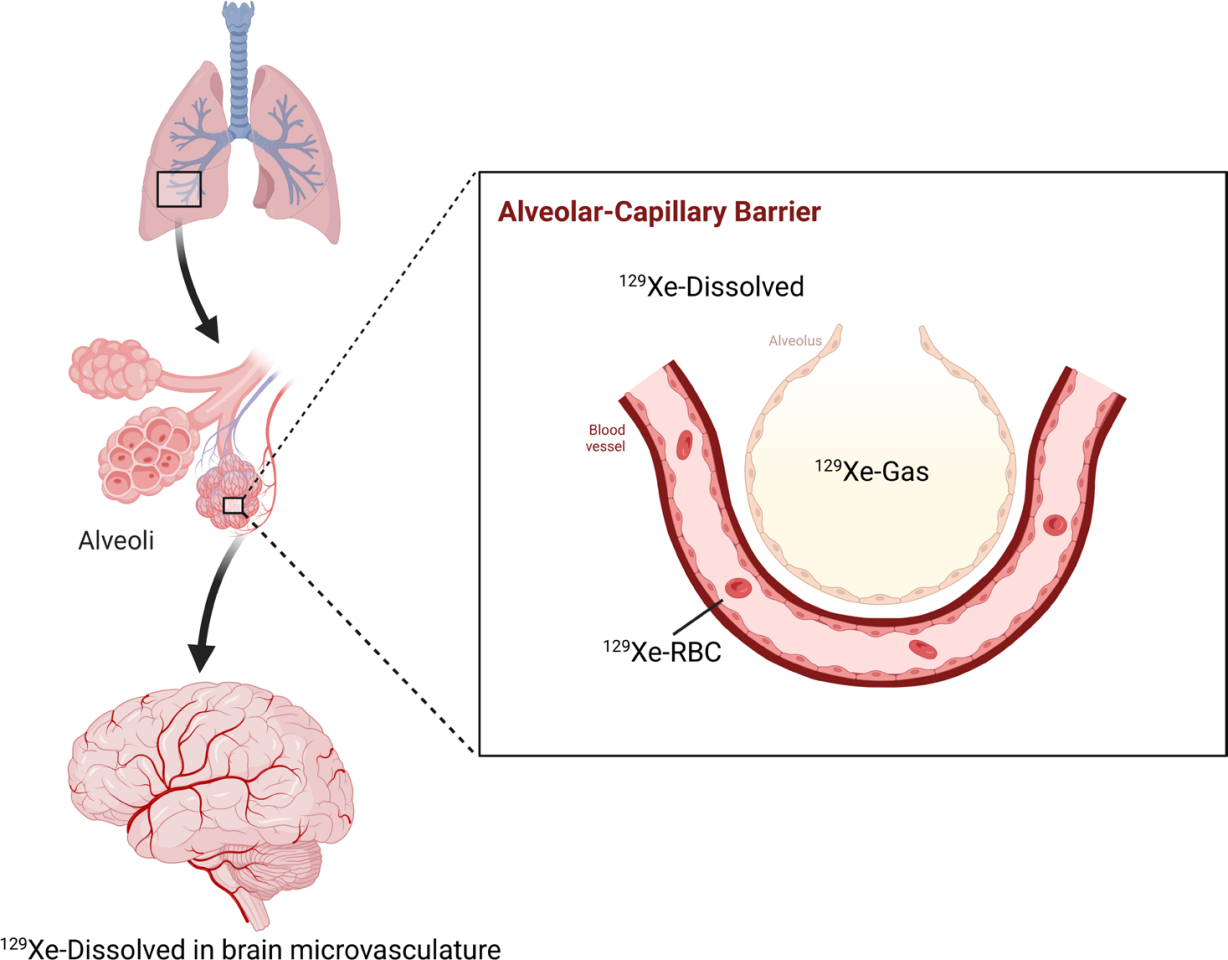
Measurement of ^129^Xe in the brain relies on the transfer of the gas from the lungs into the bloodstream and subsequently delivery to the brain vasculature.

The solubility of ^129^Xe into the blood means that perfusion‐based measurements can be made for organs other than the lungs [52]. ^129^Xe transfers from the alveoli into the bloodstream, attaching to the RBC to be delivered to organs of interest (Figure 8). While the T_1_ is shortened when in the bloodstream, limiting the SNR [32, 53, 54], measurements have shown the potential of this technique in both kidney [53] and brain measurements [54].

### 2.6 pH Measurement

pH is tightly regulated within the body, playing a vital role in physiological processes by controlling the rate of enzymatic reactions. As such, the presence of diseases causes an alteration in pH, with changes to the equilibrium often creating an acidic extracellular environment, for example, in the case of cancers and ischemia. Hyperpolarized probes offer the potential for a non‐invasive method of pH measurement.

#### 2.6.1 [1‐ ^13^C]‐Bicarbonate

The application of HP ^13^C‐bicarbonate for pH measurements relies on the pH‐dependent equilibrium between bicarbonate (HCO_3_^−^) and CO_2_. Following the injection of ^13^C‐bicarbonate, conversion and detection of CO_2_ allow for measurement of pH using the Henderson–Hasselbalch equation.

(3) $$\begin{array}{c} \mathrm{pH}={pK}_{a}+\log_{10}\left(\frac{\left[H^{13}C{O_{3}}^{-}\right]}{\left[13_{{\mathrm{CO}}_{2}}\right]}\right)\; \end{array}$$

Pre‐clinical application of hyperpolarized ^13^C‐bicarbonate has shown the ability to measure pH in the heart and in tumors [55, 56]. Whilst measurements were able to be made pre‐clinically, the short T_1_ of ^13^C‐bicarbonate (often < 10 s in vivo) limits its application in clinical use.

#### 2.6.2 [1‐ ^13^C] Pyruvate

[1‐^13^C] pyruvate is metabolically converted to ^13^C‐bicarbonate with ^13^CO_2_ as an intermediary. Therefore, detection of both bicarbonate and CO_2_ allows for pH measurement through the same method as HP ^13^C‐bicarbonate tracer. Application to the heart [57, 58, 59] and the brain [59] has shown the ability to measure pH using a clinically approved probe; however, the large chemical shift of CO_2_ alongside its low SNR limits the application in imaging.

#### 2.6.3 [1,5‐^13^C_2_] Z‐OMPD

Z‐OPMD (Z‐4‐methyl‐2‐oxopent‐3‐enedioic acid) has a pH‐sensitive chemical shift, which when hyperpolarized allows for a sensitive method of pH measurement with sufficient T_1_ for imaging and without needing to detect downstream metabolites. This probe has been applied to both phantom and preclinical murine kidney models where pH measurements have been validated for extracellular pH measurements [60].

## 3 Imaging Strategies

Once hyperpolarized probes are created, acquiring clinically useful information is guided by the imaging performed in order to optimize the data acquired. HP MR data acquisition presents unique practical challenges due to the intrinsic nature of the samples. Firstly, specialized hardware is often required, including dual‐tuned RF coils capable of transmitting/receiving at both the ^1^H frequency (for localization/anatomical reference) and the X‐nucleus frequency (e.g., ^13^C at ~1/4 the proton frequency). Secondly, hyperpolarized sequences are extremely sensitive to B0 field inhomogeneity, which can cause unacceptable signal loss or artifacts. Thirdly, there is a fundamental tension between achieving sufficient SNR, spatial resolution, spectral resolution (if applicable), and temporal resolution, all within the constrained timeframe imposed by T_1_ decay and radiofrequency RF depletion. This often necessitates compromises and sequence optimizations specific to the application. Finally, the rapid signal decay during acquisition can lead to k‐space weighting inconsistencies and potential image artifacts if not appropriately managed (e.g., through variable flip angles or specific k‐space ordering).

### 3.1 Magnetic Resonance Spectroscopy (MRS)

MRS, although not technically imaging, offers an important method for understanding hyperpolarized data. The spectroscopic method is easy to implement using an RF pulse to excite the nuclei of interest, before collecting the free induction decay (FID) to produce a spectrum of the peaks. Spectra can be localized to slices in the brain using a slice‐selective gradient. MRS offers an attractive option for metabolic measurements, allowing for metabolite peaks to be clearly visualized. Additionally, dynamic information can be produced using multiple pulse‐acquire repetitions.

### 3.2 Magnetic Resonance Spectroscopic Imaging (MRSI)

MRSI allows for spectra to be localized to a grid of voxels, providing a locally resolved spectrum for accurate visualization of metabolites. To collect spectra across a region, phase encoding gradients are often applied with slice‐selective gradients to produce a 3D MRSI image. However, the requirement for phase encoding gradients extends the time needed for imaging and, therefore, means that dynamic information is difficult to produce. Furthermore, MRSI suffers from poor spatial resolution, limiting the localized information able to be obtained. To improve MRSI, the sampling of k‐space can be altered with under sampling and k‐space center density weighted sampling reducing the overall acquisition time.

### 3.3 Echo‐Planar Spectroscopic Imaging (EPSI)

As an alternative to “conventional” MRSI, fast spectroscopic techniques such as EPSI offer a more efficient method of data acquisition. In doing so, multiple lines of k‐space can be read in one excitation using ramp sampling, but this comes at a cost of decreased SNR [61]. The direction of the k‐space readout can also be controlled with both Cartesian (i.e., line by line) and non‐Cartesian (e.g., radial and spiral).

### 3.4 IDEAL Encoding

Spectroscopic imaging can be accelerated using multi‐echo readouts, allowing k‐space to be acquired at varying TEs to encode and sample the spectral and spatial simultaneously. Spatial information is sampled along the k‐space trajectory while spectral information is sampled by repeating the same trajectory within the same T_R_ [62]. By using this imaging scheme, the number of excitations needed is reduced compared to conventional spectroscopic imaging. This can be combined with a model‐based chemical shift encoding technique to efficiently encode spectra. For this imaging method to be successful, metabolic chemical shifts and B0 must be known. In IDEAL, *n* + 1 different TEs are needed for n metabolites present and a spectrum is taken prior to imaging to obtain prior knowledge of metabolite chemical shifts [63, 64]. For hyperpolarized ^13^C pyruvate, simulations performed by Wiesinger et al. [64] showed that seven TE shifts provided a good balance between accurate chemical shift encoding while providing fast dynamic imaging.

### 3.5 Spectral Spatial (SpSp)

As an alternative to spectroscopic measurements, metabolite selective imaging can be used. Using this method, individual resonances for the metabolites of interest can be spectrally encoded using spectral‐spatial excitation [65]. The excitation pulse uses a 2D RF pulse that is slice and frequency selective, thereby allowing each metabolite to be selected individually in each slice. Following the excitation of the metabolite of interest, a spiral or echo planar readout can be applied for imaging. This offers an attractive method of imaging where quick and efficient acquisition can be applied, allowing for a customizable imaging approach for each metabolite. This allows for different spatial or temporal resolution or different flip angles to be applied to certain metabolites to maximize their respective signal. However, this requires an accurate input of the chemical shifts, which determine the excitation frequency chosen. This is further complicated by B0 field inhomogeneity or center frequency errors, where errors in setup may result in the imaging failing and no metabolites being detected.

### 3.6 Spoiled Gradient Echo (SPGR)

To obtain high resolution, single molecular images in a dynamic fashion, SPGR can be used. Through this, a low flip angle continuous sequence can be applied to generate a continuous depletion of the hyperpolarized signal. Whilst this can be used for the creation of ventilation maps [66], it is inherently inefficient for hyperpolarized imaging. Application of spoiling gradients actively destroys residual transverse magnetization after each RF pulse, failing to utilize the finite hyperpolarized state effectively compared to steady‐state sequences. This leads to faster signal depletion, making it less practical for HP imaging requiring multiple dynamics or high resolution.

### 3.7 Balanced Steady State Free Precession (bSSFP)

bSSFP offers a more efficient method of HP imaging where a train of RF pulses is applied alongside a varying gradient polarity with sufficiently short TR (TR << T_2_) that a continuous signal with varying amplitude is produced [67]. Whilst bSSFP offers a more efficient imaging scheme compared to SPGR methods, it suffers from hardware requirements where the short TR and requirement for fast gradient switching limit its application. bSSFP sequences also exhibit high sensitivity to B0 field inhomogeneities and off‐resonance effects, which can cause signal loss, banding artifacts, and complicate the separation of metabolite signals with close resonance frequencies, especially in vivo. In addition, whilst proven to be effective for single resonance hyperpolarized compounds (e.g., ^13^C‐urea or ^129^Xe ventilation maps), metabolic measurements of [1‐^13^C] pyruvate are more difficult due to close resonant of the metabolites. Nonetheless, approaches such as multi‐echo bSSFP and suppressing some metabolite peaks (e.g., ^13^C‐alanine and ^13^C‐pyruvate‐hydrate) can optimize bSSFP for metabolic measurements [68].

## 4 Data Analysis and Metrics for Hyperpolarized Imaging

Following acquisition, quantification methods of the data are needed to produce useful information. Methods of quantification depend on the HP probe and the biological question being addressed. This section covers the modeling and metrics for metabolic imaging using ^13^C HP probes and pulmonary imaging using ^129^Xe.

### 4.1 Hyperpolarized Metabolic Analysis

The acquisition from hyperpolarized metabolic studies goes much further than just producing single images of metabolic distribution. The multi‐dimensional dataset can be up to five dimensions with dynamic and spectroscopic dimensions [69]. Therefore, accurate quantification and modeling can offer both information about metabolic levels and the conversion between metabolites.

#### 4.1.1 Kinetic Modeling

Kinetic modeling seeks to use the evolution of hyperpolarized signal to model the metabolic exchange of pyruvate to other metabolites. For example, the evolution of pyruvate to lactate can be simplified to a two‐site exchange system where pyruvate and lactate are in a rate of exchange with one another. The change in the pyruvate and lactate signal can be given by the following differential equations:

(4) $$\begin{array}{c} \frac{\mathrm{d}P\left(t\right)}{\mathrm{d}t}=k_{\mathrm{LP}}L\left(t\right)-k_{\mathrm{PL}}P\left(t\right)-\mathrm{pP}\left(t\right) \end{array}$$

(5) $$\begin{array}{c} \frac{\mathrm{d}L\left(t\right)}{\mathrm{d}t}=k_{\mathrm{PL}}P\left(t\right)-k_{\mathrm{LP}}L\left(t\right)-\mathrm{pL}\left(t\right) \end{array}$$

where *P*(*t*) and *L*(*t*) are the pyruvate and lactate signal at time t, *k*_PL_ is the exchange rate from pyruvate to lactate, *k*_LP_ is the exchange rate from lactate to pyruvate and p represents the loss of polarization due to relaxation.

Rate constants, *k*_LP_ and *k*_PL_, provide the metabolic information that shows the rate of conversion of the metabolites. Several different techniques exist for modeling the kinetics. However, robust estimation of kinetic parameters using such models is often impacted by the typically low SNR of hyperpolarized data, particularly for downstream metabolites (e.g., bicarbonate, alanine). This low SNR can lead to high uncertainty in fitted rate constants. Examples include a two‐site model which requires vascular considerations offering an accurate model but comes at the cost of complexity [70]. The requirement for an arterial input function (AIF) of the injected probe is technically demanding and often impractical in clinical settings. Input‐less models avoid this but rely on simplifying assumptions about tissue perfusion and vascular input that may not always hold true. Alternatively, a two‐site input‐less model can be used to fit dynamic data timecourse data [71]. However, whilst this is easily applied, the model may fail in low SNR scenarios, which can be improved by undertaking the kinetic modeling in the frequency domain [72]. Whilst this requires a full timecourse of dynamic data (i.e., detecting the hyperpolarized ^13^C bolus arrival), it can offer a robust method of kinetic fitting.

#### 4.1.2 Ratiometric Approaches

Ratiometric approaches offer a simple method to quantify metabolism in a model‐free method. While straightforward to implement, ratiometric approaches sacrifice valuable kinetic information about absolute conversion rates. Furthermore, simple ratios can be confounded by differential T1 relaxation times, RF saturation effects across metabolites (due to different flip angles experienced or different intrinsic T1s), and delivery dynamics, potentially biasing the interpretation if not carefully considered. Nonetheless, these methods offer a simple method to measure metabolism which has been shown to correlate with biological metabolism. One such method is the “AUC ratio” [73], which uses the area under the metabolites curve to calculate a ratio. This method assumes that only pyruvate has an input function with downstream metabolites formed in the imaged organ as opposed to being delivered vascularly [69]. This was shown to be proportional to the *k*_PL_ in a two‐site, two‐way model. Alternatively, the lactate: pyruvate (*L*/*P*) peak ratio offers a measurement of the LDH metabolism against overall metabolic delivery. This therefore provides a metric proportional to the LDH concentration [69].

### 4.2 Hyperpolarized Pulmonary Modeling

HP lung imaging offers a multimodal imaging approach depending on the imaging sequence used. Ventilation maps offer a measure of HP gas distribution; however, more information can be obtained when using soluble gases such as HP ^129^Xe to measure uptake. Since ^129^Xe dissolves into the RBC and barrier tissue, measurement of the gas in the different compartments and modeling of the rate of transfer can offer greater insight into lung function. A simple method of modeling has been shown to be the ratio of RBC to barrier tissue, which has been shown to provide a global measure of gas transfer using spectroscopic data [74]. Furthermore, HP ^129^Xe signal in the RBC has been shown to oscillate because of cardiogenic oscillations [75] wherein oscillations offer a measure of the pulmonary microvascular. Whilst the RBC suffers from low SNR, measurements of the amplitude and phase changes can be used as an additional data metric for lung disease [76].

## 5 Improving the Hyperpolarized Signal

Several different approaches have been developed to improve the acquisition and analysis of the hyperpolarized signal with a focus on retaining the hyperpolarized signal. Accelerated imaging can reduce the total scan time and hyperpolarized decay by acquiring the k‐space in a quicker and more efficient manner than conventional methods. Parallel imaging (e.g., GRAPPA [77] or SENSE [78]) exploits the multi‐coil sensitivity profile to under sample k‐space in the phase encoding direction [79] offering acceleration factors of up to 4×.

As an alternative to changes in the acquisition, reconstruction, and denoising methods can be applied following imaging to increase SNR, reduce noise artifacts, and improve interpretation. Methods such as higher order single value decomposition (HOSVD) [80, 81], tensor rank truncation‐image enhancement (TRI) [82] or tensor Marchenko‐Pastur principal component analysis (tMPPCA) [83] offer up to 10‐fold improvements to the SNR. Super resolution techniques also offer a potential for clinical translation by enhancing the resolution using high resolution ^1^H images offering superior enhancement compared to traditional interpolation methods [84].

## 6 Clinical Applications

Hyperpolarized probes have been applied in vivo to investigate several clinical questions using both d‐DNP and SEOP. Currently, hyperpolarized [1‐^13^C] pyruvate and ^129^Xe have been the focus of in vivo imaging studies. HP ^13^C pyruvate and ^129^Xe have had differing impacts in the clinic, in part due to greater technological requirements for the manufacturing of the sterile injectable compounds, making the road to impact shorter for inhaled noble gases.

HP [1‐^13^C] pyruvate has shown great promise for early and more precise diagnosis and treatment monitoring in exploratory clinical trials; but has not yet reached clinical use. In comparison, ^129^Xe has begun to be used for clinical diagnosis in some regions for probing pulmonary lung disorders. The application of HP ^129^Xe in clinic is supported by both the improved/simplified and unique information offered compared to current diagnostic modalities for lung conditions.

The d‐DNP community is currently setting up the needed multi‐center clinical trials to demonstrate clinical value and thus drive the clinical adoption. The lessons learned from ^129^Xe clinical adoption should serve as a guide to drive HP ^13^C to clinical use. This section introduces the current applications of hyperpolarized imaging to the clinic, demonstrating the ability to probe disease processes.

### 6.1 Oncology

Metabolic dysfunction owing to the Warburg effect and increasing need for improved imaging methodologies has made the application of hyperpolarized imaging in cancer an attractive target. Increased conversion of pyruvate to lactate allows for the Warburg effect to be measured.

*Prostate Cancer*—The first application of [1‐^13^C] pyruvate in prostate cancer patients demonstrated the viability of the imaging method with elevated levels of lactate‐pyruvate levels in the tumor region [85, 86]. Further clinical imaging studies showed that HP [1‐^13^C] pyruvate can phenotype different grades of prostate tumors [86].

*Breast Cancer*—Application of [1‐^13^C] pyruvate to breast cancers showed the ability to differentiate between different grades with increased lactate to pyruvate ratios associated with higher grades [87]. Further studies demonstrated the ability to measure early treatment response after neoadjuvant chemotherapy, outperforming current proton MRI techniques [88].

*Renal Cancer*—Imaging of clear cell renal cell carcinoma with HP [1‐^13^C] pyruvate showed the ability to differentiate grades with greater metabolic turnover associated with more aggressive disease and potentially act as a predictor of survival [89].

**FIGURE 9:**
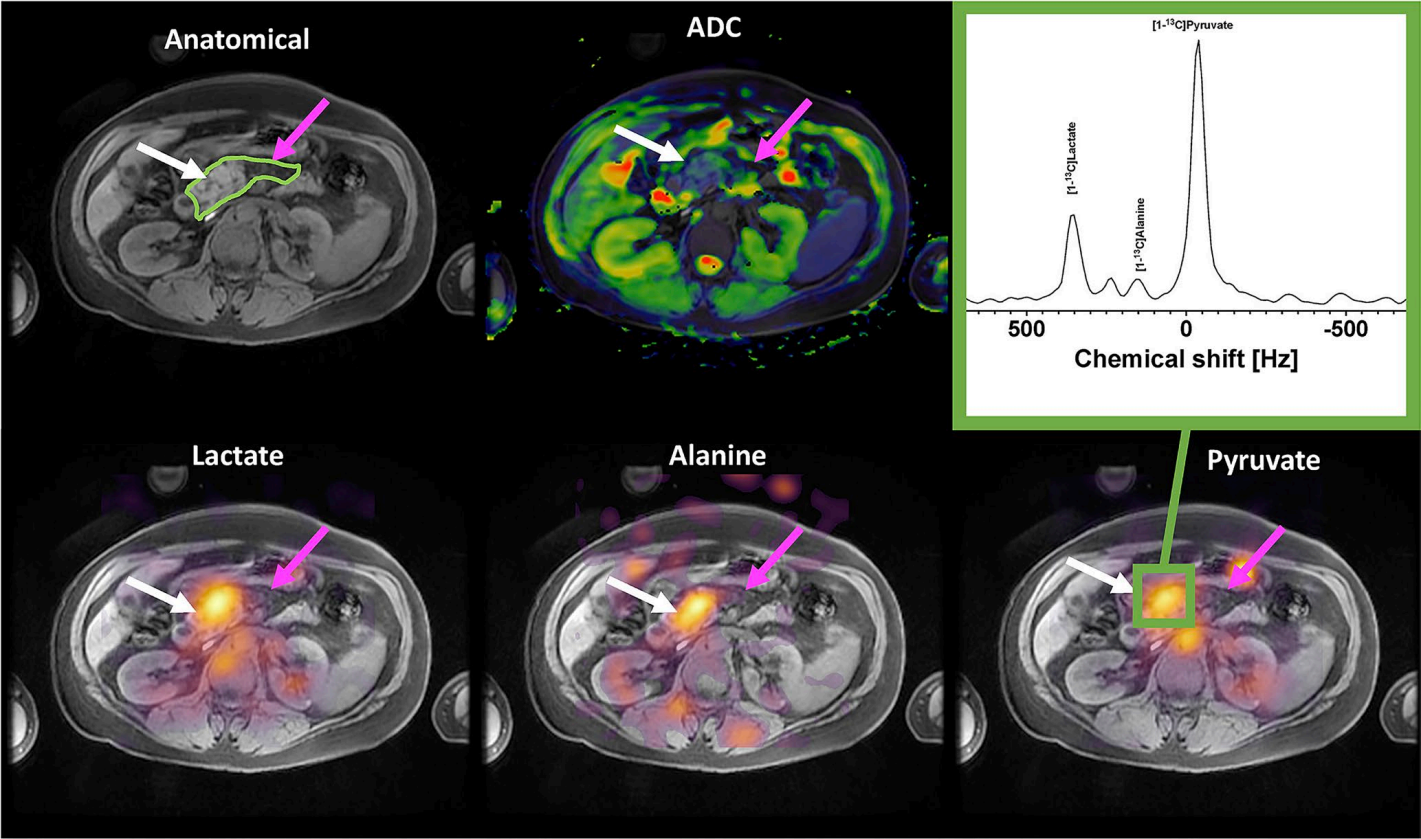
Axial slice of a pancreatic tumor in patient (white arrow) and the exocrine pancreas (purple arrow). High signal of ^13^C pyruvate, lactate and alanine were observed within the tumor as shown by the spectral extraction. Figure reproduced from reference [90] with permission from authors.

*Pancreatic Cancer—*Metabolic imaging of pancreatic cancer has shown the role that ^13^C‐alanine can play in probing oncological changes. Proof of concept work has demonstrated increased ^13^C‐alanine seen in pancreatic tumors of patients [90] (Figure 9).

*Brain Cancer*—The high metabolic activity of the brain makes downstream metabolic measurement particularly attractive since current methods to just measure glucose uptake (e.g., FDG‐PET) are limited in measuring tumor metabolism [89]. Application of [1‐^13^C] pyruvate to brain tumors revealed increased ^13^C lactate production [91] and serial HP scans showed variation of metabolic values related to progressing tumor lesions [92]. Finally, measurement of glioblastoma metabolism prior to surgical resection revealed the ability of HP [1‐^13^C] pyruvate to detect heterogeneous metabolism within the tumor alongside metabolic alterations in the contralateral hemisphere compared to healthy subjects [93].

### 6.2 Lung Disease

**FIGURE 10:**
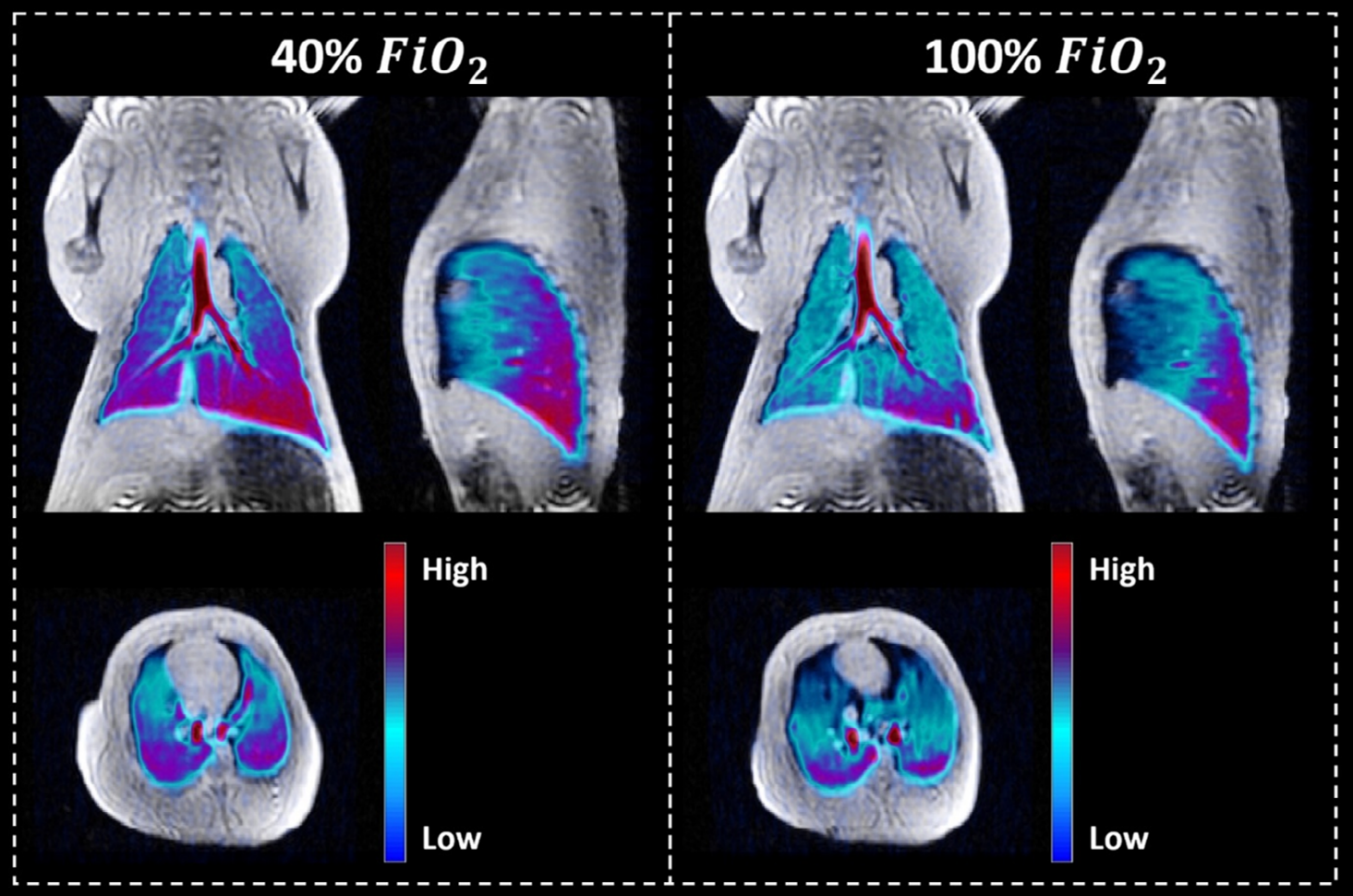
Ventilation images from a porcine lung with differing levels of fractional inspired oxygen (FiO_2_) overlaid on T_1_ proton images. Image reproduced from reference [94] with permission from authors.

**FIGURE 11:**
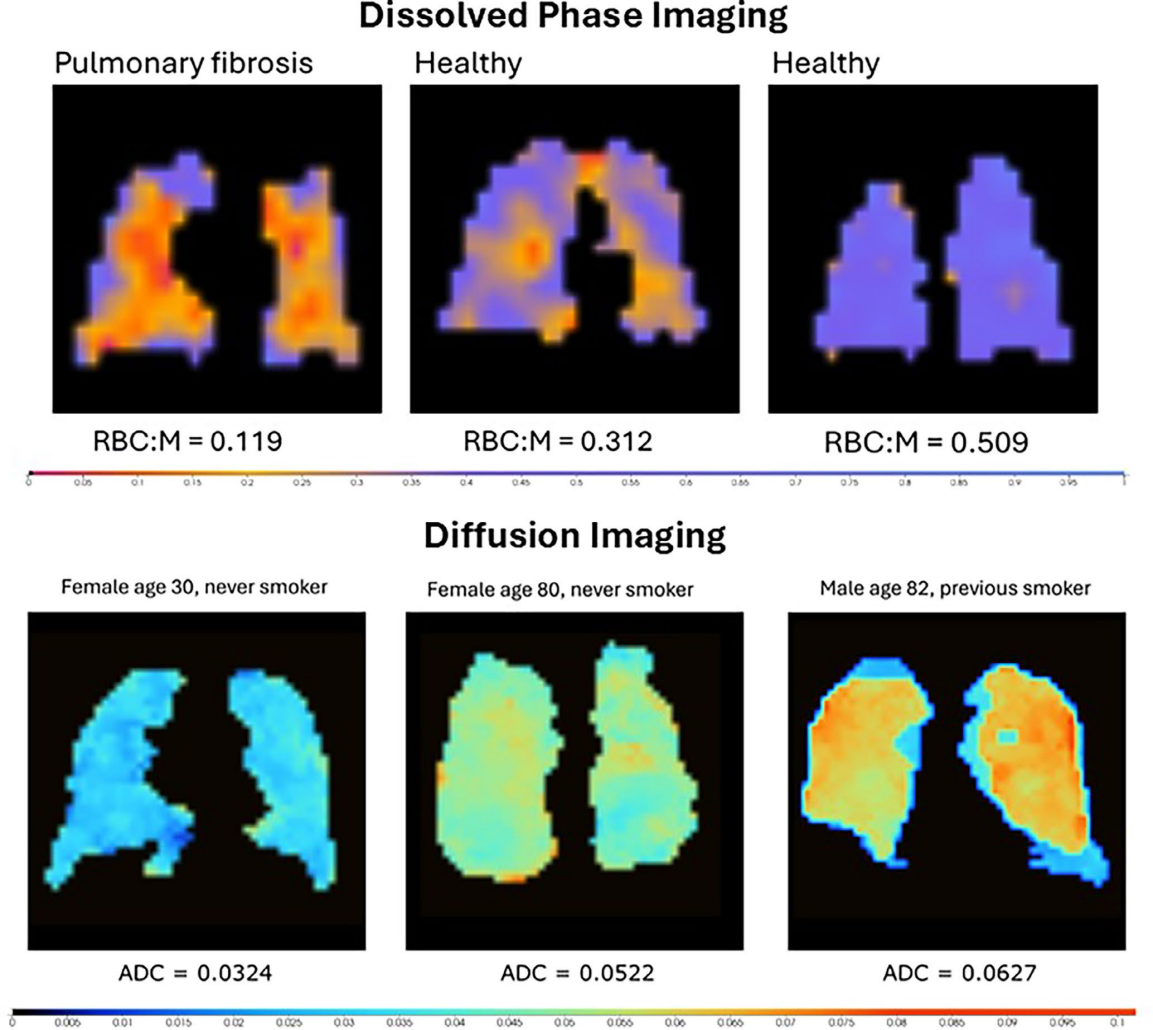
Dissolved phase and diffusion images from HP ^129^Xe in healthy and diseased lungs. Top: Dissolved phase images from a healthy volunteer and a patient with pulmonary fibrosis. The ratio of RBC to membrane (RBC:M) offers a marker of gas transfer efficiency and is impaired in patients. Bottom: Diffusion weighted images show an increase in diffusion coefficient in impaired lungs because of alveolar damage and airspace enlargement. Images provided by Dr. Mattias Kristensen.

The low proton density of the lungs means that conventional proton imaging is often limited in use for clinical information. As such, the application of a hyperpolarized gas, such as hyperpolarized ^129^Xe offers improved understanding of several clinical conditions through both ventilation imaging and uptake information of the HP gas. Ventilation images offer a measure of ventilation defects, allowing for regional lung function to be assessed. An example from a porcine lung [94] is shown in Figure 10. Further evaluation can be made using ^129^Xe uptake information due to its lipophilic properties. Notably, several lung diseases have been shown to exhibit different HP ^129^Xe properties [95]. Measurement of the impairment of gas uptake in Idiopathic Pulmonary Fibrosis (IPF) shows a reduction in ^129^Xe being extracted to the bloodstream and instead a 188% increase in barrier uptake compared to healthy controls [96]. In comparison, COPD has been shown to have a greater defect in ventilation maps [97] and reduced RBC oscillation [95]. Application to patients with post‐COVID‐19 condition showed not only abnormalities compared to healthy controls but also lung impairment that conventional imaging (such as CT) could not measure [98]. The variety of different metrics able to assess lung function offers a multifaceted imaging approach for lung disease monitoring, with Figure 11 showing the dissolved images able to be obtained.

**FIGURE 12:**
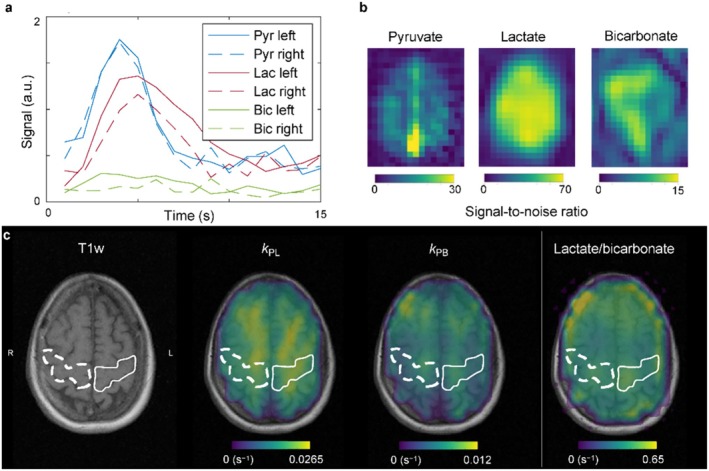
Dynamic imaging using HP [1‐^13^C] pyruvate in a patient with ALS where the left hand motor region is impaired. Signal changes over time of metabolites showed differences in regions of impairment compared to a healthy region of the brain. Increased conversion of pyruvate to lactate is seen in the left hand region because of the disease. Figure reproduced from reference [99] with permission from authors.

*Brain Disease*—Metabolic and vascular dysfunction in the brain offers novel insights for better understanding of brain disease and for improved diagnosis and treatment response. Application of [1‐^13^C] pyruvate in a one patient case study of amyotrophic lateral sclerosis (ALS) revealed metabolic alterations with increased lactate to pyruvate rates in regions of the brain associated with clinical symptoms (left motor area) [99]. Metabolic changes are shown in Figure 12, where increased conversion of lactate can be seen in the left hand motor region. Metabolic alterations were also observed in cases of traumatic brain injury (TBI) where reduced bicarbonate was seen local to the injury site [100, 101]. Application of metabolic imaging to other brain diseases has been limited to pre‐clinical studies; however, studies applied to multiple sclerosis [102], Alzheimer's disease [103, 104], and stroke models [105, 106] show changes in metabolism.

Vascular changes can be measured using HP ^129^Xe where the inhaled gas is transported via the blood to the brain for perfusion measurements. Despite the shortened T_1_ relaxation that occurs because of ^129^Xe binding to RBC, application to the brain has achieved between 20 and 40 SNR [107]. Application to a singular stroke patient revealed an area of hypo intensity corresponding to hypoperfusion because of impaired capillary delivery [108]. Finally, imaging of a small cohort of Alzheimer's disease patients (*n* = 4) revealed slower washout of xenon following inhalation compared to healthy controls, reflecting impaired perfusion in the patients brains [109].

**FIGURE 13:**
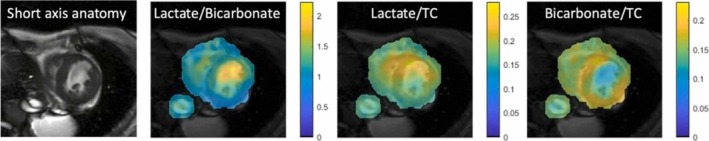
Hyperpolarized [1‐^13^C] pyruvate in a patient with heart failure 1 month prior to imaging. Reduced bicarbonate can be seen in the area of the heart where tissue death had occurred. TC = total carbon. Images reproduced from reference [110] with permission from authors.

*Cardiology*—The high metabolic activity of the heart makes it an attractive target for metabolic measurement where alterations can be detected in several heart diseases [110]. Application to diabetic patients showed impaired mitochondrial activity through a reduction of ^13^C bicarbonate levels [111]. Metabolic changes because of heart disease have been measured using [1‐^13^C] pyruvate wherein increased ^13^C lactate and decreased ^13^C‐bicarbonate levels were seen in patients with ischemic heart disease [110]. Example metabolic maps showing reduction in bicarbonate levels due to ischemia are shown in Figure 13.

## 7 Conclusion

HP MRI offers a unique imaging opportunity to enable the non‐invasive visualization of molecular processes and metabolic fluxes in vivo. Application of the technology dramatically boosts nuclear spin polarization, providing access to clinically important biological information. Several technologies exist to facilitate the production of the hyperpolarized samples with multiple probes of biological relevance. Whilst the technology suffers from several technical and logistical hurdles, the unique ability to provide dynamic insights into tissue metabolism, perfusion, and physiology offers transformative potential compared to current techniques. However, advancing the clinical adoption of HP MRI requires a shift from exploratory studies toward adequately powered clinical and multi‐center trials capable of demonstrating its clinical value in real‐world settings. Such efforts are currently underway within both the dissolution DNP (^13^C) and SEOP (^129^Xe) communities. Given the shared technological foundations and imaging workflows across hyperpolarization methods, we see an opportunity for synergistic collaboration among these communities—including those developing emerging hyperpolarization techniques—to accelerate clinical translation.
